# Transcriptomic analysis using RNA sequencing and phenotypic analysis of *Salmonella enterica* after acid exposure for different time durations using adaptive laboratory evolution

**DOI:** 10.3389/fmicb.2024.1348063

**Published:** 2024-02-27

**Authors:** Mrinalini Ghoshal, Tyler D. Bechtel, John G. Gibbons, Lynne McLandsborough

**Affiliations:** ^1^Department of Microbiology, University of Massachusetts, Amherst, MA, United States; ^2^Department of Food Science, University of Massachusetts, Amherst, MA, United States

**Keywords:** *Salmonella enterica* serovar Enteritidis, acetic acid stress, adaptive laboratory evolution (ALE), minimum inhibitory concentration (MIC), human antibiotics, mutations, genome sequencing, RNA sequencing

## Abstract

**Introduction:**

This study is the final part of a two-part series that delves into the molecular mechanisms driving adaptive laboratory evolution (ALE) of *Salmonella enterica* in acid stress. The phenotypic and transcriptomic alterations in the acid-evolved lineages (EL) of *Salmonella enterica* serovar Enteritidis after 70 days of acid stress exposure were analyzed.

**Materials and methods:**

The stability of phenotypic changes observed after 70 days in acetic acid was explored after stress removal using a newly developed evolutionary lineage EL5. Additionally, the impact of short-term acid stress on the previously adapted lineage EL4 was also examined.

**Results:**

The results indicate that the elevated antibiotic minimum inhibitory concentration (MIC) observed after exposure to acetic acid for 70 days was lost when acid stress was removed. This phenomenon was observed against human antibiotics such as meropenem, ciprofloxacin, gentamicin, and streptomycin. The MIC of meropenem in EL4 on day 70 was 0.094 mM, which dropped to 0.032 mM when removed from acetic acid stress after day 70. However, after stress reintroduction, the MIC swiftly elevated, and within 4 days, it returned to 0.094 mM. After 20 more days of adaptation in acetic acid, the meropenem MIC increased to 0.125 mM. The other human antibiotics that were tested exhibited a similar trend. The MIC of acetic acid in EL4 on day 70 was observed to be 35 mM, which remained constant even after the removal of acetic acid stress. Readaptation of EL4 in acetic acid for 20 more days caused the acetic acid MIC to increase to 37 mM. Bacterial whole genome sequencing of EL5 revealed base substitutions in several genes involved in pathogenesis, such as the *phoQ* and *wzc* genes. Transcriptomic analysis of EL5 revealed upregulation of virulence, drug resistance, toxin-antitoxin, and iron metabolism genes. Unstable S*almonella* small colony variants (SSCV) of *S*. Enteritidis were also observed in EL5 as compared to the wild-type unevolved *S*. Enteritidis.

**Discussion:**

This study presents a comprehensive understanding of the evolution of the phenotypic, genomic, and transcriptomic changes in *S*. Enteritidis due to prolonged acid exposure through ALE.

## 1 Introduction

Understanding how microorganisms adapt to harsh environmental conditions is paramount, particularly for food-borne pathogens such as *Salmonella enterica*, which pose significant threats to public health worldwide (Brown et al., 2021). Acid stress represents one such critical environmental challenge for enteric pathogens such as *Salmonella* Enteritidis, which encounter highly acidic conditions in external environments and within the host gastrointestinal tract (He et al., 2018; Kenney, 2019). During food processing, *S*. Enteritidis is exposed to acidic conditions commonly used for food preservation, flavoring, and fermentation (He et al., 2018). Additionally, *S*. Enteritidis present on food contact surfaces is often exposed to acidic cleaning agents such as organic acids (Aljumaah et al., 2020).

Analyzing how *S*. Enteritidis navigates these acidic environments is pivotal for safety and control strategies. As part of an ongoing investigation into the adaptive responses of *S*. Enteritidis to acid stress, this study constitutes the second phase of our two-part series, focusing on the comprehensive analysis of phenotypic and transcriptomic changes following adaptive laboratory evolution (ALE) of *S*. Enteritidis in acid stress. ALE entails the iterative cultivation of microbial populations under controlled stress conditions, thereby simulating natural selection in a laboratory setting (Sandberg et al., 2019). By subjecting *S*. Enteritidis to prolonged acid stress using ALE, we aim to unearth the intricate dynamics of its adaptive strategies.

In Part 1 of this series, we established the groundwork by introducing ALE as a powerful tool for investigating the adaptation of *S*. Enteritidis to acid stress. We delved into genotypic and phenotypic transformations of *S*. Enteritidis during prolonged exposure to acetic acid. ALE led to the generation of four acid-evolved lineages (EL) in our previous study, which were named EL1-EL4 (Ghoshal et al., 2023). We studied the changes in the growth rates of the ELs after prolonged and continuous exposure to acetic acid. We analyzed the changes in MIC against acetic acid and the concomitant changes in the MIC of human antibiotics in the ELs. Additionally, the presence of mutations in the form of base substitutions, insertions, and deletions was also explored, providing insights into the genetic basis of acid stress adaptation for 70 days using ALE. Building upon these findings, Part 2 of this series delves deeper into the molecular mechanisms that underpin the adaptive responses in *S*. Enteritidis through an integrated study of phenotypic transformations and transcriptomic alterations during ALE. The phenotypic changes serve as tangible manifestations of the bacterium's altered genome and physiology, while the transcriptomic shifts provide a window into the underlying gene expression patterns that drive these adaptations.

This study applies a multi-pronged approach, where we analyzed the stability of the phenotypic changes observed in the previous study (Ghoshal et al., 2023) after the removal and subsequent reintroduction of the acid stress. The ALE process was continued in EL4, and we worked on the development of another evolutionary lineage, EL5. We studied the transcriptomic changes in ELs after long-term continuous adaptation as well as the effects of short-term acid stress on the previously acid-adapted ELs.

Our goal is to present a holistic narrative of the journey of *S*. Enteritidis through adaptive evolution under acid-stress conditions, using the combined efforts of Part 1 and Part 2 of this series. By exploring both the phenotypic and molecular facets of adaptation (genetic and transcriptomic), we aspire to deepen our understanding of strategies employed by *S*. Enteritidis for survival and uncover novel perspectives for managing and combating its pathogenic effects. The culmination of this two-part series aims to provide a comprehensive resource for researchers and scientists delving into bacterial stress response and bacterial evolution under stress.

## 2 Materials and methods

### 2.1 Bacterial isolates and growth media

For this study, *Salmonella enterica* subsp. *enterica* serovar Enteritidis (ATCC BAA 1045, phage type 30) was used. Frozen stocks of the bacteria were stored at −80°C in trypticase soy broth (TSB; Sigma-Aldrich) supplemented with 15% glycerol. To revive the frozen *Salmonella* cultures, they were streaked onto tryptic soy agar (TSA; Sigma-Aldrich) and then incubated at 37°C for 18–20 h. Subsequently, single bacterial colonies were streaked onto TSA, and isolated colonies were then cultured in TSB overnight for 18 h.

### 2.2 Quantification of minimum inhibitory concentration (MIC) of acetic acid

The MIC of acetic acid against the evolutionary lineages of *S*. Enteritidis was determined using the broth dilution method (Wiegand et al., 2008) with minor modifications. Briefly, in 50 mL tubes, bacterial inoculum taken from the evolutionary lineages (EL) of 10^7^-10^8^ CFU/mL was added in 20 mL TSB containing acetic acid concentrations from 20 to 40 mM. These tubes were incubated under agitation at 37°C for ~18 h. As control measures, bacterial cultures were also introduced into TSB containing 200 mM acetic acid (negative control) and TSB without acetic acid (positive control). The lowest concentration of acetic acid that completely inhibited visible bacterial growth and exhibited OD_600_ ≤ 0.1 using the spectrophotometer was defined as MIC. This procedure was replicated three times for each lineage. The MIC of acetic acid for the ELs was assessed every 5 days during the ALE study.

### 2.3 Adaptive laboratory evolution of *S*. Enteritidis in acetic acid

Adaptive laboratory evolution of *S*. Enteritidis in acid stress was performed by continuing the method described in our previous study with some additions (Ghoshal et al., 2023). Briefly, at the beginning of the ALE study, MIC of WT (wild type) *S*. Enteritidis was quantified, and an inoculum of 10^7^-10^8^ CFU/mL was added to three 50 mL conical tubes containing 20 mL of TSB to generate the three replicates of EL1. Inoculum from the WT was also added to three tubes containing 26 mM acetic acid (sub-MIC acetic acid; MIC acetic acid for WT was 27 mM) in TSB, which served as the three replicates of EL2. The six tubes were incubated at 37°C for 18–20 h under shaking. After 20 h, the OD_600_ of the six tubes was measured and recorded, and an inoculum of 10^7^-10^8^ CFU/mL from each tube was serially transferred into six new tubes for day 2. This process was repeated till day 70 of the ALE study, and a similar inoculum was transferred to fresh tubes for each of the ELs daily. Samples from the previous day were stored at −80°C over the course of the evolutionary process. The number of bacterial generations “n” was calculated using the following equation (Lee et al., 2011):


$$\begin{array}{l} \text{n}=log\left(N/N_{0}\right)/log\left(2\right) \end{array}$$

where *N* is the final number of cells in the conical tube after 20 h at the time of passage to the next day's tubes. *N*_0_ is the initial number of cells that are transferred to each conical tube at the beginning of ALE for that day. The initial and final numbers of cells were estimated daily by measuring the OD_600_ with a spectrophotometer and using plate counts. The generation number calculation assumes that each cell is viable, the death rate is negligible, the cells are growing exponentially throughout the ALE experiment, and the cells are dividing by binary fission. The MIC of acetic acid and several antibiotics was quantified against all three replicates of EL1-EL4 every 5 days.

After ALE day 70, the continuous adaptation of evolutionary lineages EL1-EL4 was halted, and the ELs were frozen at −80°C. EL4 was recovered from freezing and adapted in TSB without acid stress for 48 h with a serial transfer at 20 h. This culture of EL4 was denoted as EL4-FR (EL4 frozen and recovered without stress). Subsequently, EL4-FR was exposed to 30 mM acetic acid for 20 h, and this culture was termed EL4-A2 (EL4 that had undergone a second exposure to acid stress). EL4-FR (three replicates) was also reintroduced into 30 mM acetic acid and adapted through the ALE process for 20 more days, and this culture was denoted as EL5. The MIC of acetic acid and several antibiotics was quantified against all three replicates of EL5 every 5 days until ALE day 90.

### 2.4 Quantification of MIC of antibiotics against *S*. Enteritidis evolutionary lineages

The MIC of the antibiotics meropenem, ciprofloxacin, gentamycin, and streptomycin were determined using MTS strips (MIC test strips, Liofilchem) (Matuschek et al., 2018; van den Bijllaardt et al., 2018). The test strips for each antibiotic had a concentration range of 0.016–256 μg/mL, and MIC was determined using the method previously described (Ghoshal et al., 2023). Briefly, overnight cultures of the various evolutionary lineages were cultivated using their respective acetic acid concentrations. The following day, OD_600_ was adjusted to ~10^7^CFU/mL in TSB. Bacterial cultures were applied onto cotton swab applicators and streaked onto TSA plates to establish uniform bacterial growth. The MTS test strips were then meticulously positioned at the center of the TSA plates using sterile forceps. Subsequently, the plates were incubated at 37°C for 18 h−20 h, and the zones of inhibition encircling the test strips were documented. The point where the zone of inhibition intersected the MIC test strip was designated as the MIC. This procedure was replicated three times for each replicate in all evolutionary lineages.

### 2.5 Bacterial whole genome sequencing

Genomic DNA extraction and sequencing were performed at Seqcenter (Pittsburgh, PA, USA). Illumina sequencing of the WT was conducted on a NextSeq 2000 sequencer, generating 151-bp paired-end reads. Afterward, low-quality reads were trimmed, and adapter sequences were eliminated from Illumina sequences using bcl-convert version 3.9.3. In addition, long sequence reads were generated through Oxford Nanopore (ONT) PCR-free ligation library preparation. These ONT reads were subject to trimming of adapters and quality assessment using porechop33 version 0.2.3_seqan2.1.1 (RRID:SCR_016967). A hybrid assembly, incorporating both Illumina and ONT reads, was generated using Unicycler version 0.4.8. The quality of the assembly was evaluated using QUAST version 5.0.2 (Gurevich et al., 2013). Subsequently, the complete genome assembly was characterized, and plasmid sequences were identified through the NCBI Nucleotide BLAST database. Gene models were predicted, and functional annotations were added using Prokka version 1.14.5 with the default parameters along with “—rfam” (Tatusova et al., 2016; Haft et al., 2018). For EL4 and EL5, the same culturing and DNA extraction procedure as that of the WT strain was employed. However, for the evolved lineages, only Illumina sequencing was used, generating 151-bp paired-end libraries following the aforementioned steps.

### 2.6 Genome assembly and identification of polymorphism

Genomic analysis of EL5 from ALE day 90 was performed using the methods from our previous study (Ghoshal et al., 2023). The genomes of EL5 on ALE day 90 and EL4 on ALE day 70 were mapped to that of the WT genome. All software mentioned below was used with default parameters unless specified otherwise. BWA version 0.7.15 (RRID:SCR_010910) was used to map EL4 and EL5 to the ancestral genome (Li, 2013). Samtools (v 1.14) (RRID:SCR_002105) was used to index the sorted BAM files, and bamaddrg was used to add read groups to the indexed, sorted BAM files (Danecek et al., 2021). Joint genotyping of the evolved strains was then performed using Freebayes (v 1.3.1) (RRID:SCR_010761) (Garrison and Marth, 2012). Low-quality site filtering was performed using VCFtools (v 0.1.14) (RRID:SCR_001235) with the following parameters: –remove-filtered-all –minQ 20 –min-meanDP 50. GATK (v. 4.0.6) (RRID:SCR_001876) was used to convert the resulting VCF files into table format (McKenna et al., 2010). A SnpEff database was built for the *S*. Enteritidis BAA-1045 WT reference genome, and SNP annotation prediction was performed in the evolved strains using SnpEff (v. 4.1) (Cingolani et al., 2012). A second variant calling software, Breseq (v. 0.35.4) (RRID:SCR_010810), was used by SeqCenter to align and compare evolved lineage sequence reads to the ancestor (Deatherage and Barrick, 2014). Assemblies were generated from the Illumina reads for each evolved lineage using SPAdes (v. 3.13.1) (RRID:SCR_000131) with the parameters “-k 21,33,55 –careful” (Bankevich et al., 2012).

### 2.7 Bacterial RNA sequencing

Samples were DNAse treated with Invitrogen DNAse (RNAse free). Library preparation was performed by SeqCenter (Pittsburgh, PA, USA) using Illumina's Stranded Total RNA Prep Ligation with Ribo-Zero Plus kit and 10bp IDT for Illumina indices. Sequencing was done on a NovaSeq 6000 with 2 × 51bp reads. Demultiplexing, quality control, and adapter trimming were performed with bcl-convert (v4.0.3) (Illumina, 2021). EL5 was grown in 30 mM acetic acid until ALE day 90, while WT *S*. Enteritidis was exposed to 26 mM acetic acid (sub-MIC of acetic acid for WT) for 18 h for RNA sequencing. Further, EL4-FR was also exposed to 30 mM acetic acid for 18 h before RNA sequencing. This sample has been described from here on as simply EL4-A2 to denote that EL4 was exposed to acid for the second time. EL5, EL4-A2, and WT were also grown for 18 h in TSB without acid stress and served as controls. Three biological replicates for each of the samples were sequenced.

### 2.8 Bacterial RNA sequencing analysis

Read mapping was performed with HISAT2 (Kim et al., 2019), and read quantification was performed using Subread's feature Counts (Liao et al., 2014) functionality. Read counts were loaded into R (R Core Team, 2020) and were normalized using edgeR's (Robinson et al., 2010) Trimmed Mean of M values (TMM) algorithm. Subsequent values were then converted to counts per million (cpm). Differential expression analysis was performed using edgeR's exact test for differences between two groups of negative-binomial counts with an estimated dispersion value of 0.1. The results of the qlfTest for all genes, in addition to the normalized cpm, were generated. The differentially expressed gene's normalized cpm were then used to create a heatmap. Genome indexing and mapping were performed using bwa (v0.7.17) with default parameters. Samtools (v1.14) was used to sort and index BAM files. Bedtools2 (v2.30.0) was used to extract read counts per transcript from gene coordinates in the sorted BAM files. The DESeq2 (v.1.38.3) package was used in R (version 4.2.3 and RStudio 2023.03.1446) to normalize read counts and identify differentially expressed genes. Differential expression thresholds were defined using log-fold change [log2(FC)] values >2 (upregulated) and <-2 (downregulated) with a *P*_Adj_ value ≤0.01. Principal component analysis (PCA) was performed on DESeq2 normalized read counts using JMP Pro (version 17) to examine the relationship between samples and replicates. For comparing the expression of the different classes of genes, annotations were generated from EggNOG and Blast2GO within the OmicsBox software (Götz et al., 2008; Cantalapiedra et al., 2021).

### 2.9 Statistical analyses

All bacterial growth measurements were biologically triplicated, and their differences were examined by a two-sided *t*-test assuming unequal variance (Welch's *t*-test). GraphPad Prism was used to generate most of the graphs in this project. Statistical significance was calculated with GraphPad Prism using two-way ANOVA. A *p* ≤ 0.05 was considered statistically significant. Fisher's exact test was used to calculate the statistical significance for the percentage of genes upregulated and downregulated as revealed in the RNA seq data.

## 3 Results

### 3.1 Adaptive evolutionary process of *S*. Enteritidis to acetic acid

*Salmonella* Enteritidis was subjected to different concentrations of acetic acid using adaptive laboratory evolution (ALE). The evolutionary lineages (EL) EL1-EL4 were adapted with daily transfers for 70 ALE days, and the ALE process till day 70 has been described in detail in our previous study (Ghoshal et al., 2023). Briefly, the minimum inhibitory concentration (MIC) of acetic acid for the wild type (WT) *S*. Enteritidis was measured to be 27 mM. Evolutionary lineages EL1 and EL2 were initiated from WT *S*. Enteritidis. EL1 was grown without exposure to acid stress, while EL2 was cultivated in 26 mM acetic acid (sub-MIC of acetic acid). Over time, the MIC of EL2 increased to 29 mM, prompting the creation of EL3, which was subsequently grown in 28 mM acetic acid (new sub-MIC of acetic acid). After 30 days, EL3's MIC further rose to 31 mM, leading to the initiation of EL4, which was cultivated in 30 mM acetic acid and subjected to daily transfers in fresh media. The ELs were grown in triplicates, and transfers were carried out to create new evolutionary lines from corresponding replicates.

**Figure 1:**
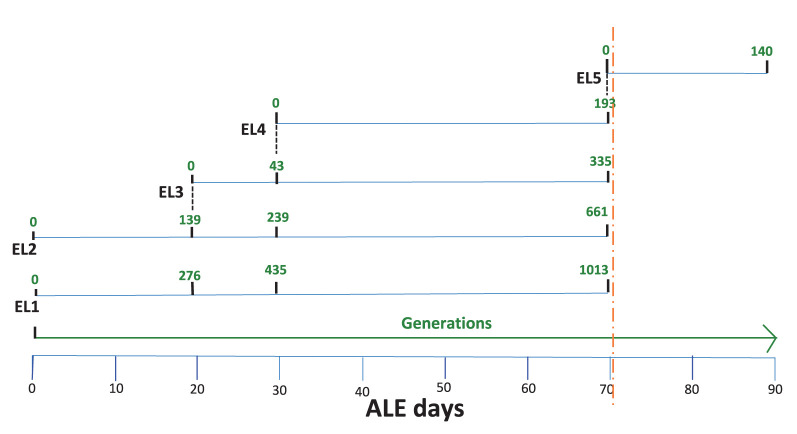
Adaptive evolution of *S*. Enteritidis evolutionary lineages (EL) in acetic acid. EL1-EL4 were adapted with daily transfers for 70 days. EL3 and EL4 were initiated from EL2 and EL3, respectively (indicated by black dotted lines). After 70 days, the evolutionary process was halted, and the ELs were frozen at −80°C (orange dotted line). Subsequently, EL4 was recovered in TSB without stress for 48 h and readapted to acetic acid until ALE day 90. This readapted culture was labeled EL5. The number of generations for each EL is shown in green.

The current study describes the ALE process after 70 days. The adaptive evolutionary process was halted after 70 days, and all the ELs were frozen at−80°C. Subsequently, EL4 was recovered from freezing in TSB without acid stress (EL4-FR) and then readapted in 30 mM acetic acid for 20 more days. This readapted evolutionary lineage was denoted as EL5. In the previous study, we analyzed several phenotypic and genomic changes that occurred in EL2, EL3, and EL4 after exposure to acetic acid and compared it to EL1, which was not exposed to any stress and served as our control (Ghoshal et al., 2023). In this study, we focused on the phenotypic, genomic, and transcriptomic changes in EL5 after exposure to acid stress until ALE day 90. The entire evolutionary process is shown in Figure 1. ALE days refers to the total number of days since the beginning of our ALE study. Experiments were conducted with EL4 from ALE day 70 (EL4-D70) and EL5 from ALE day 90 (EL5-D90). Additionally, EL4 after ALE day 70 was grown in TSB without stress for 2 days and denoted as EL4-R (EL4 recovered in TSB without acid). EL4 that was frozen at −80°C after ALE day 70 and recovered in TSB for 2 days without acid was denoted as EL4-FR (EL4-frozen and recovered without acid). The growth rate of EL1-EL4 in the presence and absence of acetic acid has been described in the previous study (Ghoshal et al., 2023). On Day 90, the bacterial growth rate of EL5 in the absence and presence of acetic acid was determined to be 0.49 and 0.36 respectively.

### 3.2 Change in MIC of acetic acid in the adapted evolutionary lineages

**Figure 2:**
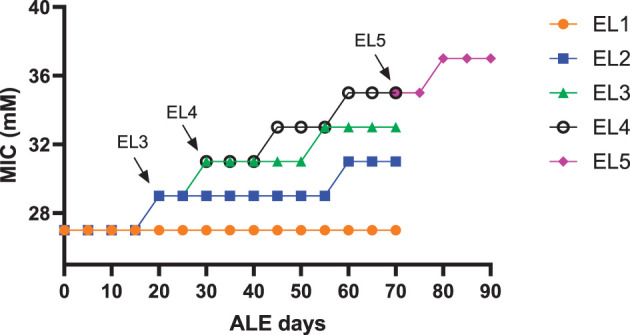
The MIC of acetic acid of the evolutionary lineages EL1-EL5. The acetic acid MICs of EL1-EL4 were measured every 5 days till day 70 of the ALE study. EL5 was initiated from EL4, and its acetic acid MIC was also measured every 5 days until ALE day 90. The arrows indicate the initiation of EL3, EL4, and EL5 from EL2, EL3, and EL4, respectively.

The acetic acid MIC of EL1-EL5 quantified during the study is shown in Figure 2. The MIC of acetic acid for EL1-EL4 was measured every 5 days as described in the previous study (Ghoshal et al., 2023). After 70 days, the MIC of EL5 was also similarly quantified every 5 days till 90 ALE days. The arrows in Figure 2 indicate the number of ALE days at which each EL was initiated from the previous one. EL5 was adapted in 30 mM acetic acid, and an increase in its MIC against acetic acid was observed on ALE day 80, which remained constant until ALE day 90.

### 3.3 Change in susceptibility to acetic acid and human antibiotics in the evolutionary lineages

We studied if freezing caused a change in the MIC against acetic acid in EL4. Acetic acid MIC of EL4 from ALE day 70 (EL4-D70), EL4-FR, and EL5 on ALE day 74 and ALE day 90 were compared. No significant differences were observed between the MIC of acetic acid in samples compared (*p* > 0.05), as shown in Table 1. This indicates that this phenotypic response to acid stress adaptation remained in the ELs even when the stress was removed, making it a stable phenotype. The same experiment was repeated with EL4-R (data not shown). Even in this case, no significant changes were observed in the acetic acid MIC of EL4-R as compared to EL4-D70 and EL4-FR. The only significant difference that was observed was between EL1-D70 (adapted in no stress for ALE day 70) and EL4-D70 (*p* ≤ 0.01).

**Table 1:** Change in susceptibility to acetic acid and antibiotics in the evolutionary lineages.

	**EL1-D70**	**EL4-D70**	**EL4-FR**	**EL5-D74**	**EL5-D90**
Acetic acid (mM)	26	35	35	35	37
Meropenem (mg/L)	0.047	0.084	0.037	0.084	0.125
Ciprofloxacin (mg/L)	0.016	0.047	0.032	0.047	0.094
Gentamycin (mg/L)	0.38	0.83	0.46	0.83	1.5
Streptomycin (mg/L)	2	6	3	6	8

Mean MIC of acetic acid, meropenem, ciprofloxacin, gentamycin, and streptomycin against EL1 and EL4 on ALE day 70 and EL5 on ALE days 74 and 90 is shown. EL1 was initiated from WT S. Enteritidis and adapted in TSB without acetic acid for 70 ALE days. EL4 was adapted in 30 mM acetic acid from ALE day 30 to ALE day 70. EL4 on day 70 was frozen at −80°C and recovered in TSB for 2 days without acid stress and is referred to as EL4-FR (EL4 from day 70 frozen and recovered without stress). Finally, EL4-FR was readapted to 30 mM acetic acid for 20 days and named EL5.

A significant increase in the MIC of various antibiotics against EL4-D70 as compared to EL1-D70 was observed for all the antibiotics tested, as seen in Table 1. We also analyzed how the MIC values against the same antibiotics changed for EL4-FR and EL5 as compared to EL4-D70. The MIC of antibiotics meropenem, ciprofloxacin, gentamycin, and streptomycin (Table 1) were studied. After recovery from freezing in the absence of stress, EL4-FR demonstrated reduced susceptibility to the human antibiotics tested. There was a significant reduction in the MIC values of all the antibiotics in EL4-FR as compared to EL4-D70 (*p* < 0.01). The MIC values of EL4-R were also similar to those of EL4-FR (*p* > 0.05, data not shown). However, after reintroduction in acetic acid, EL5 showed a recovery in the MIC values on ALE day 74 against the antibiotics, and its values were comparable to those of EL4-D70 (*p* > 0.05). Additionally, EL5 on ALE day 90 demonstrated a significant increase in the MIC values of all the human antibiotics tested as compared to EL5-D74, EL4-D70 and EL4-FR (*p* ≤ 0.01).

**Figure 3:**
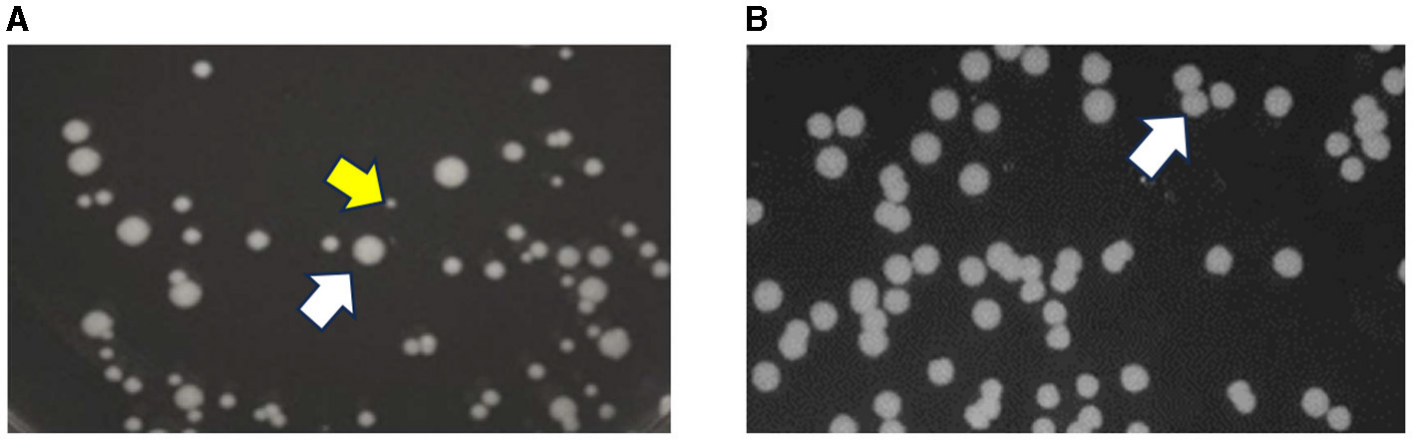
Stability of the phenotypic characteristics of EL4 before and after freezing. *Salmonella* small colony variant (SSCV) are pinpoint colonies observed in EL4 on ALE day 70 and EL5 on ALE day 90 after adaptation in acetic acid stress (yellow arrow) along with regular-sized colonies [normal colony phenotype, white arrow, **(A)**]. Homogeneous distribution of normal colony phenotype (white arrow) was observed in EL4-FR (EL4 from day 70 frozen and recovered without stress) and EL4-R (EL4 from day 70 recovered without stress) without the presence of SSCV **(B)**.

An inoculum from EL4-D70 plated on TSA showed the presence of two types of colonies: normal phenotype colonies (NPC) as well as pinpoint colonies known as *Salmonella* small colony variants (SSCV) (Figure 3A). The presence of SSCV along with NPC was also observed in EL5 on ALE days 74 and 90. However, the SSCV phenotype was not observed when the inoculum from EL4-R and EL4-FR were plated on TSA, which showed a homogeneous distribution of NPC of *Salmonella* (Figure 3B). The SSCV isolated from EL4-D70, EL5-D74, and EL5-D90 obtained after 24 h on TSA reverted back to NPC after subsequent subcultures.

### 3.4 Comparative genomics of the acid-adapted evolutionary lineages EL4 and EL5

The genomic analysis of the evolutionary lineages EL1-EL4 on ALE day 70 was performed and the results have been described in the previous study (Ghoshal et al., 2023). In the present study, we compared the genomic profile of EL5-D90 to EL4-D70, and the results are shown in Table 2. Only replicate 2 of EL5 maintained the *phoQ* mutation p.Ile421Leu from EL4. The other two replicates of EL5 lost that mutation but yielded a new *phoQ* mutation of p.Leu250Phe. Again, only replicate 2 of EL5 maintained the *thiB* mutation that occurred in all three replicates of EL4. These results suggest that replicate 2 of EL5 may have used a different pathway of adaptation than replicate 1 and 3. Additionally, the replicates of EL5 had novel mutations that were absent in EL4. Replicates 1 and 3 of EL5 showed mutations in the *spoT* gene that encodes for guanosine 3′,5′-bis(diphosphate) 3′-pyrophosphohydrolase (ppGppase), which is an enzyme that catalyzes the hydrolysis of guanosine tetraphosphate (ppGpp) to guanosine triphosphate (gtp) (Magnusson et al., 2005). Additionally, frameshift mutations were observed in the *wzc* gene in replicates 1 and 3 of EL5-D70, which is involved in capsule formation in *E. coli* (Yang et al., 2021).

**Table 2:** Mutations in EL5 after adaptation in acetic acid for 90 ALE days were identified using genomic analysis and compared to EL4 after adaptation in acetic acid for 70 ALE days.

**Position**	**Gene**	**Mutation**	**Ancestor allele**	**Evolved allele**	**Protein Product**	**EL4**	**EL5**
1,745,485	*rfbE*	Missense	A	T	CDP-paratose 2-epimerase	R1: p.Asn220Tyr R2: p.Asn220Tyr R3: p.Asn220Tyr	
1,972,868	*phoQ*	Missense	T	G	Virulence sensor histidine kinase PhoQ	R1: p.Ile421Leu R2: p.Ile421Leu R3: p.Ile421Leu	R2: p.Ile421Leu
1,973,381	*phoQ*	Missense	G	A	Virulence sensor histidine kinase PhoQ		R1: p.Leu250Phe R3: p.Leu250Phe
3,776,974	*thiB*	Missense	AACGGTGACGGTGA	AACGGTGACGGTGACGGTGA	Thiamine-binding periplasmic protein	R1: p.Val193_Thr194dup R2: p.Val193_Thr194dup R3: p.Val193_Thr194dup	R2: p.Val193_Thr194dup
439,516	*tuf1*	Synonymous	C	T	Elongation factor Tu 1	R3: p. His320His	
518,130	*oadB*	Synonymous	T	C	Oxaloacetate decarboxylase beta chain	R3: p. Leu85Leu	
1,477,186	*[ackA]–[hxpA]*	Deletion	Δ2,207 bp	x	Acetate kinase- Hexitol phosphatase	R1: Δ2,207 bp R2: Δ2,207 bp R3: Δ2,207 bp	R1: Δ2,207 bp R2: Δ2,207 bp R3: Δ2,207 bp
103,404	*spoT*	Missense	C	A	ppGpp (guanosine 3′-diphosphate 5-′ diphosphate)		R1: p.Ser368Ile R3: p.Ser368Ile
1,715,212	*wzc*	Frameshift	GCCCGCA	GCCGCA	putative tyrosine kinase		R1: p.Pro455fs R3: p.Pro455fs
2,024,345	*nimT*	Synonymous	C	T	CynX/NimT family MFS transporter		R1: p.Leu151Leu R3: p.Leu151Leu
4,453,078	*yiiM*	Synonymous	G	A	3-alpha domain		R1: p.Asp131Asp R3: p.Asp131Asp

All the evolutionary lineages were mapped to the parent genome of *Salmonella enterica* serovar Enteritidis BAA 1045. R1, R2, and R3 refer to the three replicates in each evolutionary lineage.

### 3.5 Determining the transcriptomic profile of EL5 in the presence of acetic acid stress

RNA-sequencing and differential gene expression analyses were performed on EL4 and EL5 to investigate how ALE affected their transcriptomic profile in the presence of acetic acid over time. The transcriptomic profile of EL5 (grown in 30 mM acetic acid until ALE day 90) and EL4-A2 (described in Section 2.2) (grown in 30 mM acetic acid for 18 h) was compared to that of WT *S*. Enteritidis, which was exposed to 26 mM acetic acid (sub-MIC of acetic acid for WT) for 18 h before RNA sequencing. EL5, EL4-A2, and WT were also grown for 18 h in TSB without acid stress and served as controls. Three biological replicates for each sample were sequenced.

**Figure 4:**
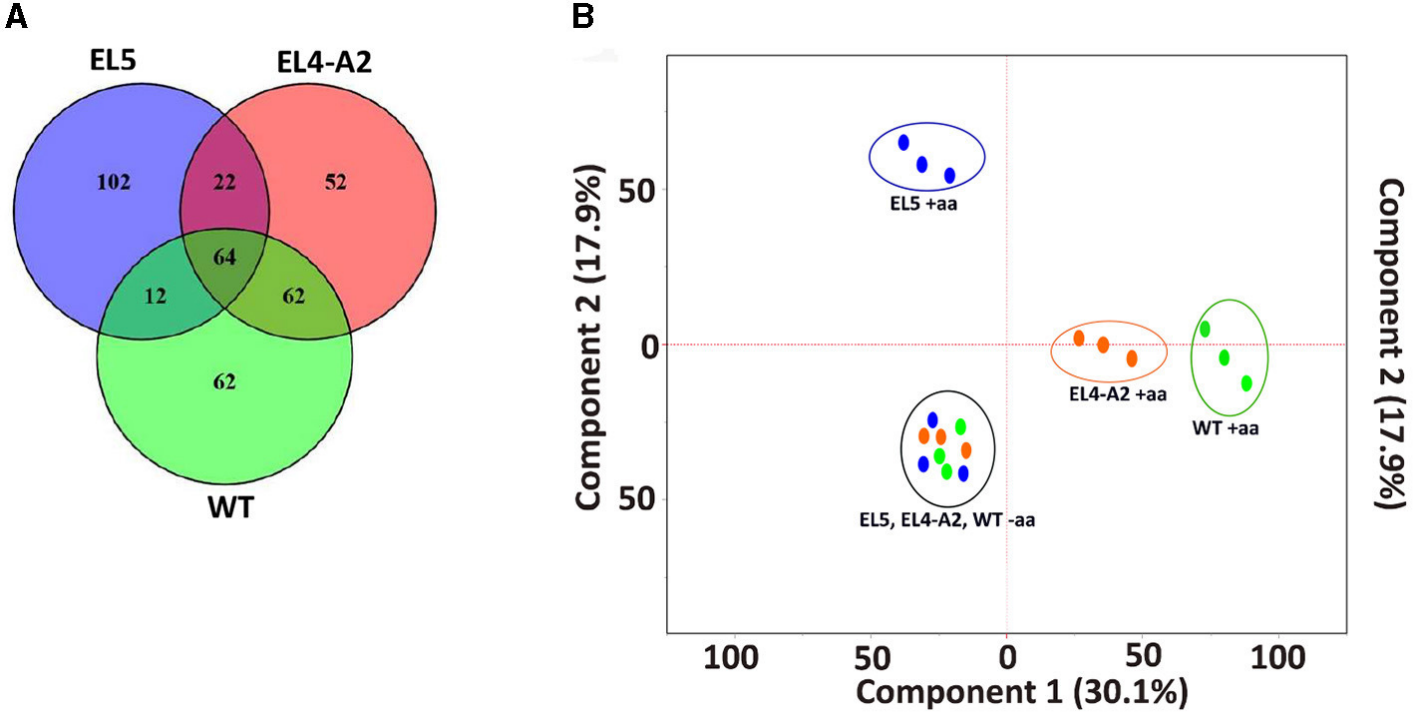
Similarities between the transcriptomic profiles of EL5, EL4-A2, and WT *S*. Enteritidis after growth in acetic acid. A Venn diagram of the top 200 upregulated genes in acetic acid stress is shown in **(A)**. PCA plot of the gene expression profiles of EL4, EL5, and WT *S*. Enteritidis with and without acetic acid stress **(B)**. The blue dots inside the blue circle are the three replicates of EL5 after treatment with 30 mM acetic acid until ALE day 90, the orange dots inside the orange circle are EL4-A2 adapted to 30 mM acetic acid for 20 h, and the green dots inside the green circle are WT *S*. Enteritidis grown in 26 mM acetic acid for 20 h. EL4-A2 refers to the EL4 that was exposed to acid stress the second time for 20 h. The blue, orange, and green dots inside the black circle are the three replicates of EL5, EL4-A2, and WT, respectively, grown in TSB for 20 h without acid stress. Here, “+aa” and “–aa” refer to the presence and absence of acetic acid, respectively.

The Venn diagram in Figure 4A shows 200 of the highest upregulated genes in EL4-A2, EL5, and WT with log_2_foldchange ≥2 and *p*_adj_ ≤0.01. About 64 of the 200 genes were upregulated in all three lineages, most of which were related to cellular physiological functions such as elongation factor Tu (EF-Tu) and glutathione synthase (*gshB*). However, some of the genes were related to stress response, such as the small toxic protein IdrD and alternative sigma factors RpoS and RpoH. While 22 of the same genes were upregulated in EL5 and EL4-A2, 62 of the same genes were upregulated in EL4-A2 and WT, and only 12 of the same genes were upregulated between EL5 and WT. EL5 had the highest percentage of genes that were exclusively upregulated, without being upregulated in EL4-A2 or WT. The transcription profiles of the specific genes that were found mutated in EL1-EL4 in our previous study were analyzed in EL5 and EL4-A2 in acetic acid and compared to that of WT in acetic acid (Table 3).

**Table 3:** Change in expression of specific genes in EL5 and EL4-A2 as compared to WT in acetic acid after ALE day 90, which were found to be mutated in EL1-EL4 after ALE day 70.

**Gene**	**Mutation**	**EL with the mutation**	**Protein Product**	**EL5 vs. WT**	**EL5 vs. EL4-A2**	**EL4-A2 vs. WT**
*ptsP*	Missense	EL1, EL2	Phosphoenolpyruvate-dependent phosphotransferase system	+ 3-fold	+ 2-fold	No change
*nimT*	Synonymous	EL1, EL2	2-nitroimidazole transporter	No change	No change	No change
*barA*	Missense	EL2	Signal transduction histidine-protein kinase BarA	+ 3-fold	+ 3-fold	No change
*alaS*	Missense	EL2	Alanine–tRNA ligase	+ 2-fold	+ 2-fold	No change
*phoQ*	Missense	EL3, EL4, EL5	Virulence sensor histidine kinase PhoQ	+ 2.3-fold	+ 2-fold	No change
*phoP*	Missense	EL2	Virulence transcriptional regulatory protein PhoP	No change	No change	No change
*zinT*	Frameshift	EL2	Metal-binding protein ZinT	−2-fold	−2.5-fold	No change
*ybaY*	Frameshift	EL2	putative lipoprotein YbaY	N/A	N/A	N/A
*fhuA*	Missense	EL2	Ferrichrome outer membrane transporter/phage receptor	No change	No change	No change
*rpoB*	Missense	EL2	DNA-directed RNA polymerase subunit beta	+ 2.5-fold	+ 2.3-fold	No change
*rfbE*	Missense	EL4	CDP-paratose 2-epimerase	No change	No change	No change
*thiB*	Missense	El4, EL5	Thiamine-binding periplasmic protein	N/A	N/A	N/A
*tuf1*	Synonymous	EL4	Elongation factor Tu 1	+ 3-fold	+ 3.6-fold	No change
*oadB*	Synonymous	EL4	Oxaloacetate decarboxylase beta chain	N/A	N/A	N/A
*wzc*	Frameshift	EL5	Putative tyrosine kinase	No change	No change	No change
*spoT*	Missense	EL5	ppGpp (guanosine 3′-diphosphate 5-′ diphosphate)	N/A	N/A	N/A
*nimT*	Synonymous	EL5	CynX/NimT family MFS transporter	N/A	N/A	N/A
*yiiM*	Synonymous	EL5	3-alpha domain	N/A	N/A	N/A

EL5 was exposed to acetic acid for 90 ALE days; EL4-A2 and WT were exposed to acetic acid for 18 h before RNA sequencing; ‘+' and ‘-' refer to up and down-regulation of the genes. At least a 2-fold change is considered to be change. N/A indicates that the specific genes in question did not show up in our RNA sequencing profile.

The principal component analysis (PCA) of genome-wide DeSeq2 normalized gene counts for WT, EL4-A2, and EL5 is shown in Figure 4B. The PCA results reveal independent clustering of WT (green circle), EL4-A2 (orange circle), and EL5 (blue circle) replicates in acetic acid, which suggests distinct expression profiles between these three groups when grown under acid stress. All three replicates of EL4-A2, EL5, and WT grown in TSB were found to cluster together (black circle). Figures 4A, B demonstrate that when compared to WT in acetic acid, EL5 exhibits greater transcriptomic variation than EL4-A2. Information about the RNA sequencing alignment and RNA sequencing statistics have been provided in Supplementary Tables S1, S2, respectively.

**Figure 5:**
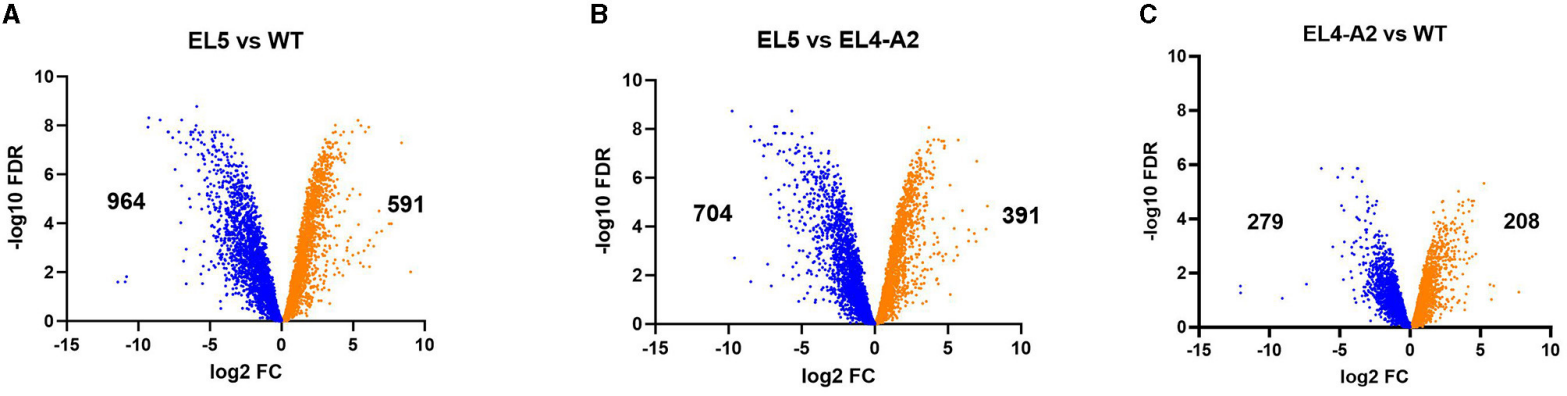
Volcano plots showing the overall changes in gene expression profiles between EL5 and WT **(A)**, EL5 and EL4-A2 **(B)**, and EL4-A2 and WT **(C)** *S*. Enteritidis. EL5 was grown in 30 mM acetic acid for 90 ALE days, EL4-A2 was exposed to 30 mM acetic acid for the second time for 20 h, and WT was grown in 26 mM acetic acid for 20 h. For each comparison, the downregulated and upregulated genes are shown in blue and orange, respectively.

Volcano plots showing the global changes in the expression of genes between WT, EL4-A2, and EL5 are shown in Figure 5. Genes having a minimum of two-fold upregulation or downregulation in their expression levels (*p*_adj_ value ≤ 0.01) have been characterized as differentially expressed. At a global level, many genes appear to be significantly upregulated and downregulated in acetic acid in EL5 as compared to the WT (Figure 5A) and EL4-A2 (Figure 5B). A total of 591 genes were upregulated, and 964 genes were downregulated in EL5 vs. WT in acetic acid stress. Further, 391 genes were upregulated, and 704 genes were downregulated in EL5 vs. EL4-A2. The differential expression of genes in EL4-A2 compared to WT was also statistically significant (p_adj_ value ≤ 0.01) (Figure 5C), with 208 genes being upregulated and 279 genes downregulated in EL4-A2 vs. WT.

**Figure 6:**
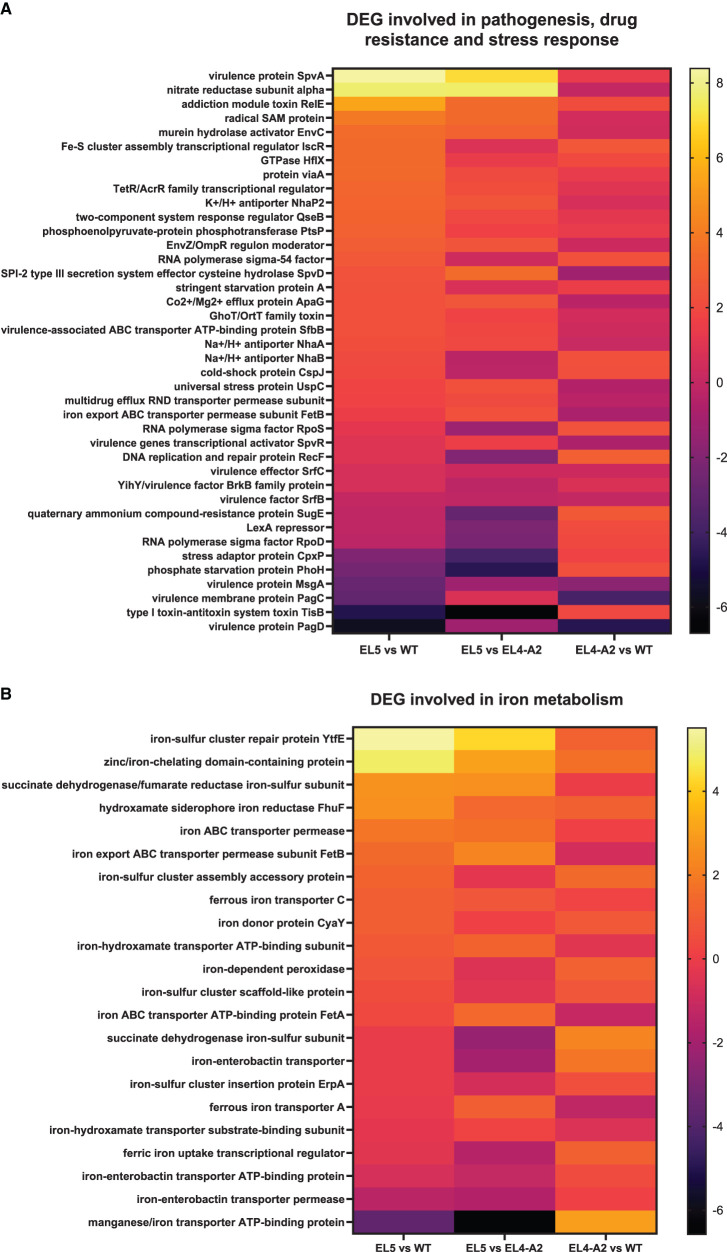
Heat maps demonstrating the differential expression of several classes of genes in EL5, EL4-A2, and WT *S*. Enteritidis after treatment with different concentrations of acetic acid. Differentially expressed genes (DEG) involved in pathogenesis, drug resistance, and stress response are shown in **(A)**. The differential expression of genes involved in iron metabolism is shown in **(B)**. EL5 was grown in 30 mM acetic acid for 90 ALE days, EL4-A2 was exposed to 30 mM acetic acid for the second time for 20 h, and WT was grown in 26 mM acetic acid for 20 h.

Heat maps demonstrating the relative expression profiles of various genes involved in pathogenesis, stress response, and drug resistance after exposure to acetic acid stress are depicted in Figure 6A. A large fraction of these genes was found to be upregulated in EL4-A2 and EL5 with respect to WT. A few notable genes that were significantly upregulated in EL5 and EL4-A2 vs. the WT were acid shock protein and Na^+^/H^+^ antiporter NhaB, which is known to help regulate intracellular pH levels. Some of the genes that were upregulated in EL5 vs. both EL4-A2 and WT were virulence protein SpvA, nitrate reductase subunit alpha, addiction module toxin RelE, and radical SAM protein. Virulence protein SpvA and toxin RelE were found to be upregulated more than 8-fold and 6-fold, respectively, in EL5 with respect to the WT (p_adj_ ≤ 0.01). Three genes that were significantly downregulated in EL5 and EL4-A2 vs. WT were the virulence membrane protein PagC, phage virulence protein, and virulence protein PagD. Further, virulence protein MsgA was significantly downregulated in EL5 vs. both EL4-A2 and WT.

Several genes involved in iron metabolism were also differentially expressed in EL4-A2, EL5, and WT during acetic acid stress (Figure 6B). Most of the iron metabolism genes were upregulated in EL5 and EL4-A2 vs. the WT. A large number of these genes encode for iron transporters, especially ATP Binding Cassette (ABC) transporters. A high expression of iron transporters has been linked to the increased virulence of *S*. Enteritidis (Domínguez-Acuña and García-Del Portillo, 2022), indicating that EL4-A2 and EL5 might have higher virulence capabilities as compared to WT in acetic acid. These results indicate that ALE in acetic acid stress has the potential to increase virulence in *S*. Enteritidis. One significant observation was that a gene coding for a manganese/iron transporter ATP-binding protein was significantly downregulated in EL5 vs. EL4-A2.

**Figure 7:**
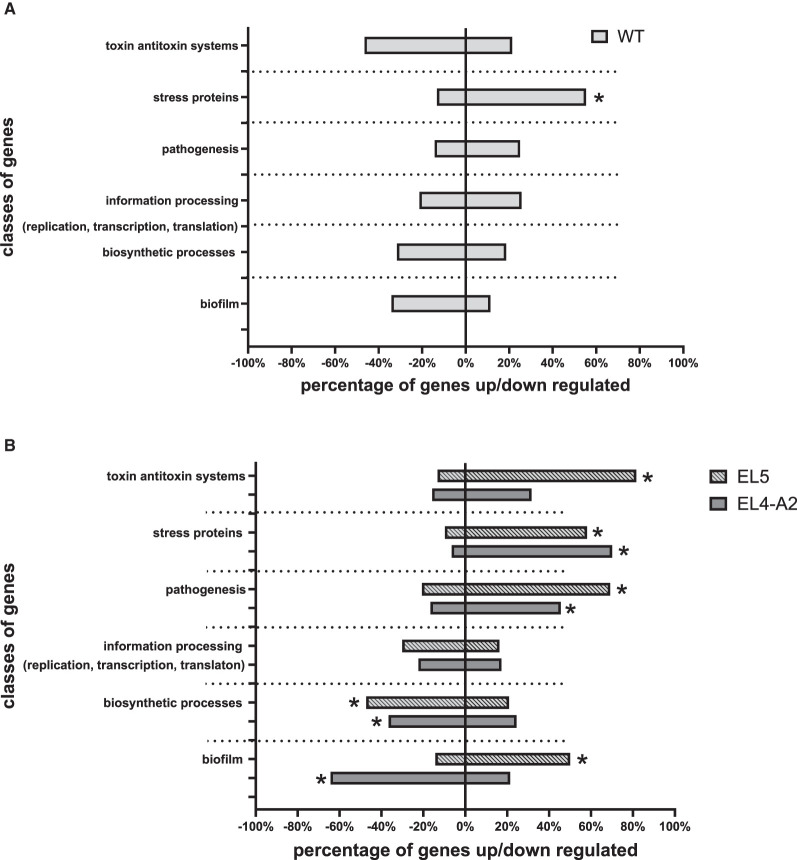
Overview of differentially expressed genes in EL5 vs. WT and EL4-A2 vs. WT according to functional categories after exposure to acetic acid. Changes in the expression of genes of WT grown in 26 mM acetic acid with respect to WT grown in TSB without acetic acid are shown in **(A)**. Changes in the expression of genes in EL4-A2 and EL5 grown in 30 mM acetic acid as compared to WT grown in 26 mM acetic acid are shown in **(B)**. The ‘*' in **(A)** refers to a statistically significant (*p* < 0.01) change in the expression of genes of a specific functional category in WT grown in 26 mM acetic acid with respect to WT grown in TSB without acetic acid. The ‘*' in **(B)** refers to a statistically significant (*p* < 0.01) change in the expression of genes of a specific functional category in EL4-A2 and EL5 grown in 30 mM acetic acid as compared to WT grown in 26 mM acetic acid.

In the presence of acid stress, WT showed an upregulation of stress proteins as compared to WT without acid stress (Figure 7A). The expression of different classes of genes in EL5 and EL4-A2 in acetic acid were compared to WT in acetic acid in Figure 7B. EL5 in acetic acid demonstrated an increase in the expression of TA systems, pathogenesis, as well as biofilm-forming genes and downregulation in the expression of genes involved in biosynthetic processes as compared to WT in acetic acid. EL4-A2 in acid demonstrated an increase in the expression of genes involved in stress and pathogenesis while showing a downregulation in the expression of genes involved in biosynthetic processes and biofilm formation as compared to WT in acetic acid.

## 4 Discussion

This study explored the interplay between ALE of *S*. Enteritidi*s* under acid stress and its subsequent impact on susceptibility to acetic acid and human antibiotics. We studied if the bacteria can quickly regain their previously acquired adaptations upon stress reintroduction, providing insights into the reversibility and persistence of stress-induced changes. Freezing and reviving EL4-D70 in a neutral growth medium (TSB without acetic acid) allowed us to attribute any observed responses in EL5 primarily to the reintroduction of the stressor (acetic acid) rather than the effects of residual acetic acid from previous exposure. Although EL4 and EL5 shared some common genetic adaptations due to acid stress, labeling the reintroduced population (EL5) as a new EL also added clarity to our ALE study, acknowledging the potential complexity of adaptive processes.

The increase in acetic acid MIC against EL5 over time under acid stress aligns with that of the other ELs (EL2-EL4), indicating a consistent trend from EL2 to EL5 (Figure 2). Several other studies have also demonstrated that bacterial populations can evolve and adapt in response to prolonged exposure to specific stressors (Li et al., 2019; Sulaiman and Lam, 2020). Although the antibiotic MICs increased in EL4-D70, EL5-D74, and EL5-D90, all except ciprofloxacin remained within susceptible ranges as defined by established critical breakpoints (FDA, 2023). Elevated MICs suggest a shift toward reduced susceptibility, which is a potential precursor to future resistance. EL5 exhibited intermediate resistance to ciprofloxacin (FDA, 2023) on ALE day 90, indicating ongoing adaptation to acid stress. This is indicative of a correlation between the mechanisms of acid stress adaptation and ciprofloxacin adaptation and underscores the importance of understanding *Salmonella* acid adaptation to prevent reduced antibiotic efficacy.

The increase in acetic acid MIC in the ELs suggests the acquisition of genetic changes that confer a selective advantage under acidic conditions. This has also been described in our previous study where, after exposure to acetic acid for 70 ALE days, the ELs had acquired several genetic mutations in the form of base substitutions and deletions (Ghoshal et al., 2023). Building on these findings, our study explored the effects of removing acid stress on antibiotic MIC. The reversible nature of the observed changes in MIC against the antibiotics (Table 1) is intriguing. EL4 was recovered in TSB without acid stress for 2 days (EL4-R), and EL4 frozen and recovered in TSB without stress for 2 days (EL4-FR) exhibited significantly reduced antibiotic MICs compared to EL4-D70 (*p* ≤ 0.01). This suggests that adaptation without acetic acid may have resulted in a loss of the antibiotic tolerance acquired during the initial adaptation to acetic acid. While stress adaptation provides short-term benefits, maintaining them in the absence of stress might be energetically costly or less compatible with optimal growth and functioning (Windels et al., 2020). EL4-R and EL4-FR prioritized growth over maintaining high antibiotic MICs, reflected in increased daily OD_600_ values of EL4-R and EL4-FR compared to EL4-D70 (*p* < 0.01; data not shown). This aligns with findings in *E. coli* after the removal of initial stress (Gottesman et al., 2009; Dunai et al., 2019), emphasizing the dynamic nature of bacterial responses to environmental pressures and their effects on antibiotic susceptibility. The observed reduction of MICs against antibiotics in the absence of acid stress also suggests that stress adaptation might involve specific transcriptional changes contributing to increased antibiotic MICs in the presence of acid stress. The rapid restoration of MICs in EL5 upon acid stress reintroduction (Table 1) indicates high phenotypic plasticity in the adapted ELs. It also implies retained adaptive changes even in the absence of continuous stress, suggesting stable genetic alterations.

The genomic analysis of EL5, as compared to EL4, revealed the presence of several mutations, such as mutations in the *phoP/Q* system, which help in acid adaptation (Table 2) (Lang et al., 2021; Han et al., 2023). This possibly explains why the increased acetic acid MIC of EL4-D70 was not lost in EL4-R or EL4-FR upon stress removal, and no significant differences in their acetic acid MIC values were observed (*p* > 0.05; Table 1). EL5 acquired a *spoT* gene mutation, potentially altering (p)ppGpp response, resulting in increased stress tolerance. (p)ppGpp is a signaling molecule that helps in bacterial survival during stress (Pacios et al., 2020) and causes large-scale transcriptional changes by binding directly to the RNA polymerase in gram-negative bacteria (Irving et al., 2021). The level of stress tolerance in *E. coli* is dependent on the amount of (p)ppGpp available in the cells (Spira and Ospino, 2020). We hypothesize that mutations in the *spoT* gene indirectly contributed to higher antibiotic resistance, as the stringent response by (p)ppGpp might affect various cellular processes, including those relevant to antibiotic susceptibility. EL5 also showed a mutation in the *wzc* gene, which is involved in capsule formation (Yang et al., 2021). Mutations in the *wzc* gene have been reported to provide resistance against antibiotics in *E. coli* (Jazdarehee et al., 2017). These changes in the capsule structure might limit the penetration of antibiotics, thereby conferring resistance to antibiotics. The mutations in *spoT* and *wzc* genes in EL5 could synergistically enable EL5 to resist human antibiotics more effectively compared to EL4-D70. This could possibly explain why we observed an increase in the antibiotic MIC values in EL5-D90 as compared to EL4-D70, indicating further adaptation in acid stress (Table 1). Some of the mutations observed in EL4-D70 were not present in EL5-D90, as shown in Table 2. This could possibly be because these mutations were not homogenous throughout the EL4-D70 cultures and were subsequently lost during the propagation of EL5.

RNA-sequencing was performed on EL4 and EL5 to study the transcriptional changes caused during adaptation to acetic acid stress over varying durations. EL4-A2, representing the combined effects of prior long-term acid adaptation and short-term acid adaptation, was used, allowing us to investigate the interplay between pre-adaptation and short-term stress response. The PCA plot (Figure 4B) demonstrates that the expression profile of EL5 in acetic acid was more distantly related to those of EL4-A2 and WT in acetic acid. This indicates that EL5 possibly has more unique mechanisms for coping with acetic acid stress than EL4-A2 and WT. This could be attributed to the longer continuous ALE of EL5 in acid as opposed to EL4-A2 and WT. The volcano plots in Figure 5 indicate that EL5 exhibits significant downregulation of numerous genes (*p* ≤ 0.01), potentially due to a high number of persistor cells. We observed unstable *Salmonella* small colony variants (SSCV) in EL4 and EL5 (Figures 3A, B), consistent with findings in other studies highlighting their instability (Kahl et al., 2016; Li et al., 2016). SSCV are known to be persister cells that are less susceptible to antibiotics and environmental stresses than their wild-type counterparts (Proctor et al., 2006). For instance, acetic acid stress induces persistence in *E. coli* (Kawai et al., 2018). Persistence can be triggered during stress, and toxin-antitoxin systems are known to be the effectors of bacterial persistence under environmental stresses (Bakkeren et al., 2020; Jurenas et al., 2022). Evidence of toxin-encoding genes being upregulated in acetic acid stress was observed in EL5 as compared to WT (Figure 7). The Gho/Ort family toxin, toxin RelE, was upregulated in EL5 vs. WT, and the type 1 TA system toxin TisB was upregulated in EL4-A2 vs. WT (Figure 6). The Gho/Ort family toxins—RelE toxin and the TisB toxin —are known to promote persistence and tolerance to antibiotics in bacteria (Singh et al., 2010; Wang and Wood, 2011; Wang et al., 2012; Edelmann and Berghoff, 2022). The outer membrane virulence proteins SpvA and SpvD (a cysteine hydrolase) are a part of the *spvABCD* operon that is involved in bacterial pathogenesis (El-Gedaily et al., 1997; Grabe et al., 2016) and were significantly upregulated in EL5 vs. WT.

Proteins conferring antibiotic resistance, such as quaternary ammonium compound resistance protein SugE, were upregulated in EL4-A2 vs. WT, and the role of these proteins in antibiotic resistance has been reported in earlier studies (Jiang et al., 2020). EL4-A2 demonstrated a higher upregulation of drug resistance genes than the WT, indicating that short-term acid exposure, especially in lineages with prior acid exposure, also promotes drug resistance in *S*. Enteritidis. Heat maps (Figures 6A, B) reveal the two-component system EnvZ/OmpR was found to be upregulated in EL5 vs. WT in acetic acid stress. It is known to play an important role in *E. coli* and *S*. Enteritidis survival under acid stress and cellular acidification during osmotic stress (Kenney, 2019). Stress response kinase A and universal stress protein UspC were highly upregulated in EL5 vs. EL4-A2 and WT in acetic acid. The Usp family of proteins is known to be upregulated in the presence of acids and antibiotics and promote virulence in *S*. Enteritidis and *E. coli* (Liu et al., 2007; Luo et al., 2023). The two-component response regulator QseB, which promotes the expression of various virulence genes in *S*. Enteritidis (Weigel and Demuth, 2016), was upregulated in EL5 vs. WT and EL4-A2. The increase in expression of OmpR/EnvZ, UspC, and QseB, which are involved in stress adaptation and antibiotic resistance (Su et al., 2007; Viveiros et al., 2007; Deng et al., 2013; Zhu et al., 2023), possibly indirectly contributed to reduced antibiotic susceptibility in the ELs.

The upregulation of Na^+^/H^+^ antiporters, which regulate intracellular pH levels in EL5 and EL4-A2 vs. WT, indicates its possible involvement in bacterial survival under acidic conditions (Xu et al., 2014). The expression levels of the Na^+^/H^+^ transporter and K^+^/H^+^ antiporter in EL5 were higher than those of EL4-A2 and WT. Efflux pumps that transport various compounds, including different classes of antibiotics outside cells, contribute to bacterial multidrug resistance (El Meouche and Dunlop, 2018; Pandey et al., 2020). Upregulation of efflux pumps, which leads to the expulsion of antibiotics outside cells, contributed directly to an increase in antibiotic MIC values in the ELs. A multidrug RND efflux transporter and TetR/Acr transcriptional regulator were upregulated in EL5 as compared to EL4-A2. Resistance nodulation cell division (RND)-type efflux transporters provide resistance against various antimicrobial compounds in gram-negative bacteria (Matsuo et al., 2013). The TetR-family transcriptional regulators (TFTRs), such as AcrR, regulate multidrug efflux systems and stress response in *S*. Enteritidis (Colclough et al., 2019). This indicates that long-term adaptation to acid stress, as in the case of EL5, results in increased antibiotic resistance compared to shorter-term adaptation, even if previously exposed to acetic acid for a long time (as in EL4-A2).

Some genes were significantly downregulated in EL5 and EL4-A2 compared to the WT, including virulence membrane protein PagC and PagD, which are controlled by the PhoP/Q operon and MsgA. These proteins play a crucial role in macrophage survival and protecting *S*. Enteritidis from host complement-mediated killing (Nishio et al., 2005; Zhao et al., 2008; Bahramianfard et al., 2021). It is possible that in the absence of a host, the expression of several genes required for host immune evasion was downregulated, and instead, the bacteria prioritized the upregulation of genes required for survival in the current stress conditions. The RNA seq results provide insights into the transcriptomic responses during the adaptation of *S*. Enteritidis to different durations of acid stress.

The adaptive laboratory evolution of *S*. Enteritidis in acetic acid has significant implications for bacterial environmental responses. The increased acetic acid MIC reflects enhanced acid tolerance, which is critical for bacterial survival in acidic food industry settings. Changes in antibiotic MIC suggest cross-resistance, potentially impacting treatment efficacy. Genomic and transcriptomic changes in stress adaptation, virulence, and drug resistance pathways underscore bacterial complexity, potentially enhancing persistence and affecting food safety. The study highlights the bacteria's evolutionary potential under stress and emphasizes the importance of examining microbial responses to various environmental stresses. Together with our previous study, it offers a detailed view of the adaptations of *S*. Enteritidis during varied acetic acid exposures.

## 5 Conclusion

In conclusion, this study provides important insights into the process of adaptive evolution in bacterial populations and underscores the intricate relationship between stress adaptation, antibiotic resistance, and bacterial fitness. The reversible nature of antibiotic resistance highlights the adaptability of bacterial populations and the potential consequences of stress-induced adaptations. Transcriptomic analysis demonstrated the upregulation of drug resistance, virulence, iron metabolism, and stress adaptation genes during continuous acid stress. The results from the genomic analysis in our current and previous study and transcriptomic analysis in this current study highlight the importance of a comprehensive understanding of the adaptation of bacteria to stressful environments for the development of effective treatment strategies. The implications of these findings extend beyond fundamental microbiology, emphasizing the need for a holistic approach to understanding bacterial responses to stress and their impact on public health and food safety.

## Data availability statement

The RNA data generated in this project is deposited in the NCBI repository under BioSample accession numbers SAMN37104682 – WT replicates (aa and TSB); SAMN37188996 – EL4-A2 replicates (aa and TSB); and SAMN37188997 – EL5 replicates (aa and TSB). Here ‘aa' refers to the presence of acetic acid and ‘TSB' refers to the absence of acetic acid, the samples were grown in trypticase soy broth.

## Author contributions

MG: Conceptualization, Data curation, Formal analysis, Funding acquisition, Investigation, Methodology, Software, Validation, Writing – original draft, Writing – review & editing. TB: Data curation, Formal analysis, Methodology, Software, Writing – review & editing. JG: Methodology, Project administration, Software, Supervision, Writing – review & editing. LM: Conceptualization, Funding acquisition, Methodology, Project administration, Resources, Supervision, Visualization, Writing – review & editing.
