# Supplementary material for: Transcriptomic analysis using RNA sequencing and phenotypic analysis of Salmonella enterica after acid exposure for different time durations using adaptive laboratory evolution
Source: Front Microbiol. 2024 Feb 27;15:1348063. doi: 10.3389/fmicb.2024.1348063 (PMC10929716; doi:10.3389/fmicb.2024.1348063)
Supplement: Supplementary file 1 [file Table_1.pdf]

Supplementary Table 1: RNA sequencing allignment of EL5, EL4-A2 and WT of *Salmonella* Enteritidis

Sample	Total Reads	All Mapped Reads	CDS Mapped Reads	rRNA Mapped Reads	tRNA Mapped Reads
EL5_R1 in aa	32900602	32390994	9117131	25543	93832
EL5_R2 in aa	21307734	19433448	4675933	51022	25693
EL5_R3 in aa	19490166	17999101	4382697	24550	19518
EL5_R1 in TSB	31206496	30907003	7761540	144558	36953
EL5_R2 in TSB	32991240	32725091	7575838	74001	36600
EL5_R3 in TSB	29957154	29649957	6463747	109316	30191
EL4-A2_R1 in aa	26908690	26023988	1190807	104355	20208
EL4-A2_R2 in aa	38303086	37433333	6348687	74093	37252
EL4-A2_R3 in aa	38549294	38259351	4483195	114334	36711
EL4-A2_R1 in TSB	30187662	29894895	5072879	14387	15138
EL4-A2_R2 in TSB	31861044	31702586	7625148	66162	24491
EL4-A2_R3 in TSB	32179938	32016261	7142387	128168	63750
WT_R1 in aa	25335530	23953065	546306	24409	17648
WT_R2 in aa	25438566	24489450	956767	19455	12014
WT_R3 in aa	30112848	28433498	3592905	72391	15443
WT_R1 in TSB	34178890	33885861	6647602	57419	32544
WT_R2 in TSB	32624854	32326323	9504986	13448	21833
WT_R3 in TSB	32952068	32648477	9233414	23733	23389

Footnote: The transcriptomic profile of EL5, which was grown in 30mM acetic acid until ALE day 90, and EL4-A2 was grown in 30mM acetic acid for 18 hours was compared to that of WT S. Enteritidis which was exposed to 26mM acetic acid (sub-MIC of acetic acid for WT) for 18 hours before RNA sequencing. EL5, EL4-A2 and WT were also grown for 18 hours in TSB without acid stress, which served as controls. ‘R1’, ‘R2’, ‘R3’ refers to the three replicates of each sample. ‘aa’ refers to the presence of acetic acid and ‘TSB’ refers to the absence of acetic acid, the samples were grown in trypticase soy broth.

Supplemetary table 2: RNA sequencing statistics of EL5, EL4-A2 and WT of *Salmonella* Enteritidis

Sample Name	Total Read Pairs	Total Reads (R1 + R2)	Total bp > Q30	% bp > Q30	R1 md5sum	R2 md5sum
EL5_R1 in aa	16450301	32900602	3943196014	92.375	9f8e49774c916190d6b0e688d4812922	42dfe7da72e998ff4d566aa438826f00
EL5_R2 in aa	10653867	21307734	2336186742	90.611	e71eae8e0824c0249f0e97e3ffc1e558	17e4e84a888e89ad306357540bef7e9b
EL5_R3 in aa	9745083	19490166	2244457317	92.453	4998ca996e0f058922137e18cc5413b4	61489635035f1dbc1ce14d7df5ef3e46
EL4-A2_R1 in aa	13454345	26908690	2997193087	93.269	c282b3166698db7521b417d792c2a217	5ba7ab5bc5b40be5dc1d347d6ddd1500
EL4-A2_R2 in aa	19151543	38303086	4392757726	93.46	d9138bd279432e3ee5afac2250ab434e	f3b2918c93f30c59ffd9ae82da97835b
EL4-A2_R3 in aa	19274647	38549294	4523648741	93.739	ed0cea2c99ac733bb2fb44b1d716ff44	994c2012a5cf0630fc4e821135209dcb
WT_R1 in aa	12667765	25335530	2686306873	92.506	d76be819341a44e88cdab3015e7c25ad	b4e5014d1eb817811e969a8deed81795
WT_R2 in aa	12719283	25438566	2740440085	93.176	eecf173710e3ad733c7ab847640c468e	774b1d8bd5e7fbaff2bd227f16834b6e
WT_R3 in aa	15056424	30112848	3241200321	93.099	a36a89d6038aab7a2c7545227e2f4fea	32add37c4eb3b32f396d4e0315995a87
EL5_R1 in TSB	15603248	31206496	3920678328	93.415	5cb3961dde5d712cf78bb5aa6585ebba	48b204478d8ca0247ea12181d71f5fea
EL5_R2 in TSB	16495620	32991240	4061860171	93.712	c75322d0f05fa1b01dcddecc6f3c9146	d49d9c5c29cf56e9ec6a95abda0c9ce2
EL5_R3 in TSB	14978577	29957154	3633137959	93.405	0c25858b4edf0dfddea7615a08371fe5	d048f83ddc3c9f3d832c5ca9c546bf22
EL4-A2_R1 in TSB	15093831	30187662	3554847003	93.914	2cab8e420f544c48062db5d900c67229	375d8cdc353e84c154442d4db235de8e
EL4-A2_R2 in TSB	15930522	31861044	3806936478	93.703	ea56e664743f8d70bf4d35d00ed1d376	97e8e10e6691a4f0468828c2764da781
EL4-A2_R3 in TSB	16089969	32179938	3918683758	93.699	4a622d3f13038d72e36ad6e2823eee48	56b616eb268d691fdf0f8c37fb1ca75c

WT_R1 in TSB	17089445	34178890	4127056374	93.667	e4481d49fb3b7d40350ba6ff9a6db6c9	29a3fa2418201db699f440a932db26d8
WT_R2 in TSB	16312427	32624854	3987271024	93.126	ab2ea4dc8c0aaa595df7282bfc5974b7	ce76e67b59d507143186376e62a974a4
WT_R3 in TSB	16476034	32952068	4019982890	93.636	cb3d419c62fc4edb8e6cf43b22d02cca	e4a1becd6479d69eebbc55c55a877d7f

Footnote: The transcriptomic profile of EL5, which was grown in 30mM acetic acid until ALE day 90, and EL4-A2 was grown in 30mM acetic acid for 18 hours was compared to that of WT S. Enteritidis which was exposed to 26mM acetic acid (sub-MIC of acetic acid for WT) for 18 hours before RNA sequencing. EL5, EL4-A2 and WT were also grown for 18 hours in TSB without acid stress, which served as controls. ‘R1’, ‘R2’, ‘R3’ refers to the three replicates of each sample. ‘aa’ refers to the presence of acetic acid and ‘TSB’ refers to the absence of acetic acid, the samples were grown in trypticase soy broth.
